# Supplementary figures and images for: UV light and abrasion’s role in degrading plasticulture films
Source: PLoS One. 2026 Mar 16;21(3):e0344790. doi: 10.1371/journal.pone.0344790 (PMC12991252; doi:10.1371/journal.pone.0344790)

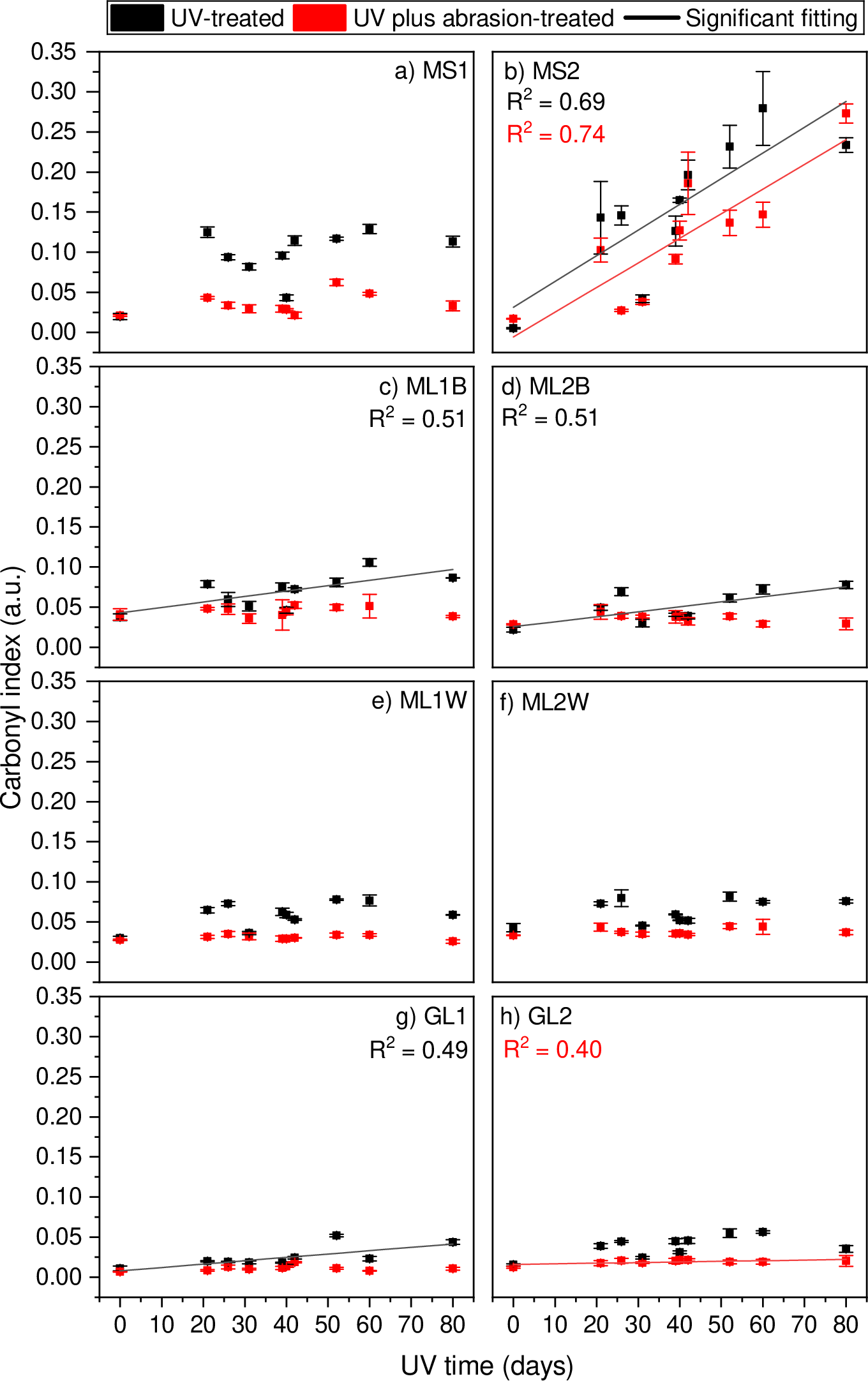

Supplement: S1 Fig — For an overview of the abbreviation, please see Table 1 in the manuscript. (TIF) [file pone.0344790.s001.tif]

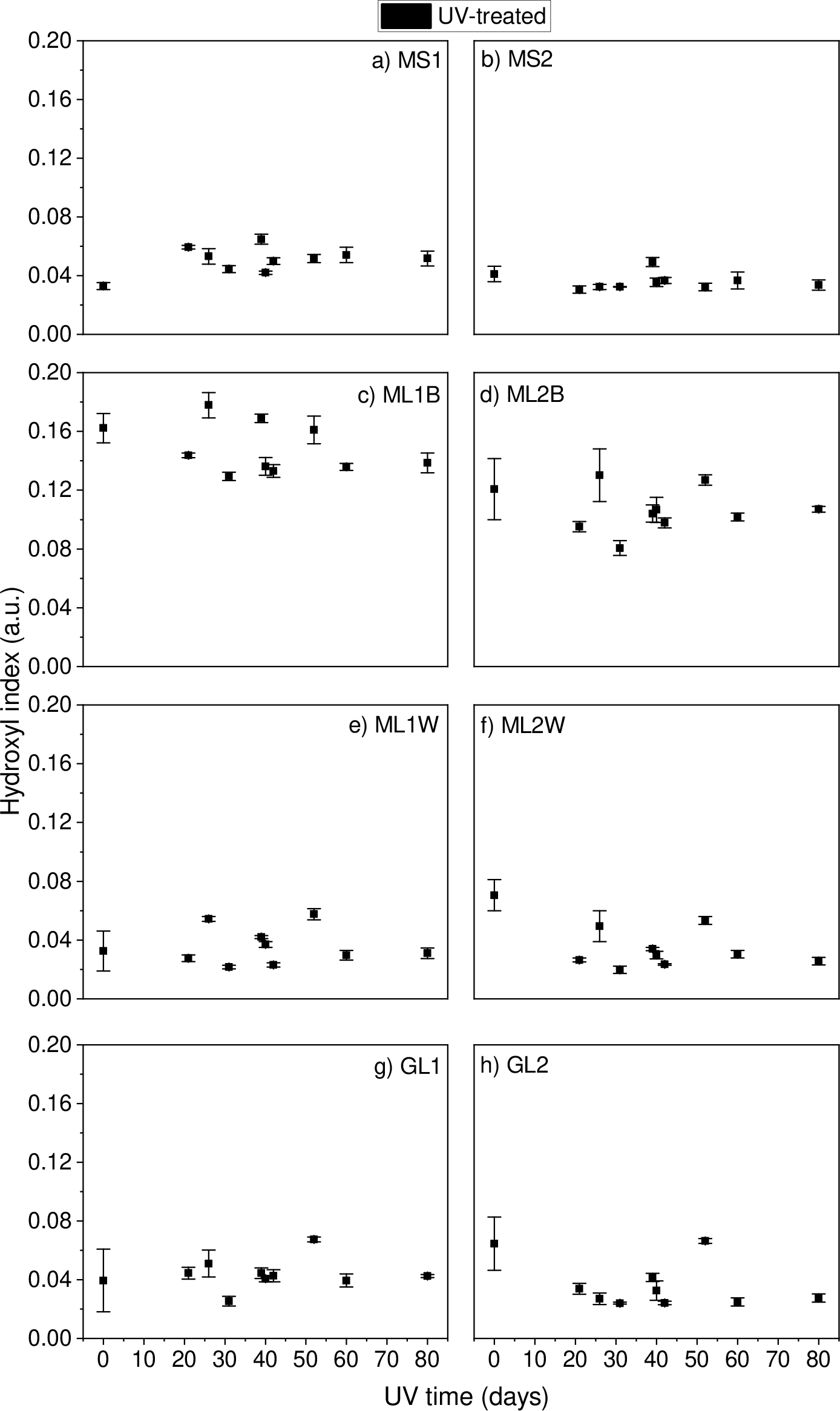

Supplement: S2 Fig — For an overview of the abbreviation, please see Table 1 in the manuscript. (TIF) [file pone.0344790.s002.tif]

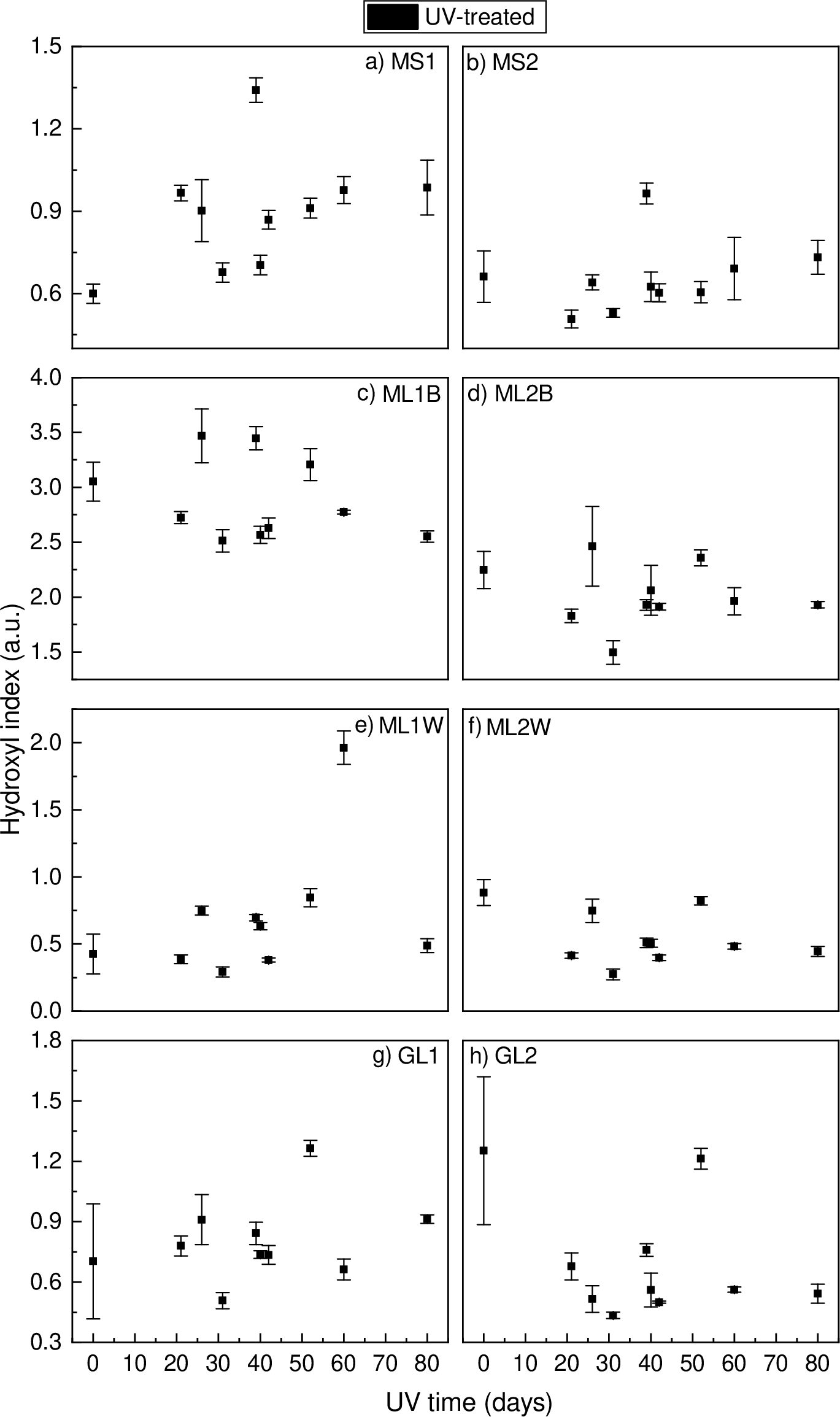

Supplement: S3 Fig — For an overview of the abbreviation, please see Table 1 in the manuscript. (TIF) [file pone.0344790.s003.tif]
