# Supplementary material for: UV light and abrasion’s role in degrading plasticulture films
Source: PLoS One. 2026 Mar 16;21(3):e0344790. doi: 10.1371/journal.pone.0344790 (PMC12991252; doi:10.1371/journal.pone.0344790)
Supplement: Attachment — Submitted filename: response_to_reviewers.docx [file pone.0344790.s005.docx]

Dear Editor,

We are pleased with the reviewer's constructive feedback and enthusiasm about this study. We appreciate the attention to detail, time, and effort invested in improving our work. The comments were taken into consideration and carefully addressed.

Our detailed answers (in italic) are presented below each reviewer's comment (bold). Line numbers here refer to the revised manuscript with track changes.

Point-by-point response – Reviewer 1

**This is an interesting manuscript that addresses a relevant topic, and the technical aspects are sound, and the interpretations presented by the authors are generally convincing. The overall aim of the study and its main findings are clearly conveyed, and the topic itself has sufficient relevance for the readership of the journal. While the level of novelty is moderate, the work can still make a meaningful contribution provided that the presentation and technical details are carefully refined.**

**The overall structure of the manuscript is reasonable, with a logical flow from introduction to conclusion. The methodology appears appropriate for addressing the stated research questions, and no fundamental methodological flaws are evident based on the current description. The discussion largely reflects the results, although clarity and readability could be improved in several places. The conclusion section would benefit from a clearer synthesis of the main findings, along with a discussion of limitations and directions for future research.**

**At this stage, I believe the manuscript is potentially suitable for publication after revision. The requested changes are mainly related to consistency, formatting, clarity, and completeness, but addressing them carefully will significantly improve the quality and professionalism of the manuscript.**

**Specific Comments**

**L4: Please check whether the reference numbering style used here is appropriate for this journal. If references are not meant to be indicated as superscripts, then this numbering style is acceptable, but it must be applied consistently throughout the entire manuscript.**

*According to the journal's guidelines, “PLOS uses the reference style outlined by the International Committee of Medical Journal Editors (ICMJE), also referred to as the “Vancouver” style.” The references on lines 28, 29, 35, 39, 40, 41, 42, 302, 305, and 321 were updated accordingly.*

**L10: When the same unit is repeated for a series of values, it is sufficient to indicate the unit only once at the end of the series. Please revise accordingly.**

*We appreciate the attention to detail. We changed the manuscript accordingly (line 10).*

**L57: Please specify where the material or equipment was purchased. The location and the company name should be clearly stated.**

*We updated the manuscript:*

*On line 57: “In this experiment, commercially available LDPE films were used* *(Firmenich, Germany), which are commonly applied as short-term…”*

*The roller used for the abrasion test was converted from a roller grill. We provide the details about the device as follows:*

*Line 103-104: “…(RG11, Roller Grill, France)…”*

**L60: When indicating numerical ranges, please use an en dash instead of a hyphen.**

*We appreciated the comment, and the manuscript was changed accordingly. The range numbers on lines 60, 61, 88, 200 and in Table 2 were updated with an en dash.*

**L66, L67: Please revise “meter” to the SI unit symbol “m” and ensure consistent usage throughout the manuscript.**

*The word “meter” was updated to the SI unit symbol “m” on lines 67 and 68.*

**L82: The dates listed here appear rather arbitrary. Please clarify the rationale or criteria used for selecting these particular dates.**

*We based our study on different levels of UV radiation exposure (low, medium, and high) across different locations in Europe. For clarification, we change the text as follows:*

*Line 82-84: “The time the samples spent in the chamber* *was set to simulate nine semi-randomised UV exposure times (21, 26, 31, 39, 40, 42, 52, 60, and 80 UV days), reflecting the variety of UV exposure conditions found in Europe.”*

**L125: Please insert commas for numbers at the thousand level (e.g., 1,000) and apply this formatting consistently throughout the manuscript.**

*We followed the suggestion and adjusted the numbers on lines 129, 154, 200, and in Table 2 accordingly.*

**L136: For temperature units in °C, the unit is usually written immediately after the number without a space. If this style is adopted, please ensure consistency across the entire manuscript.**

*We appreciate the comment. The space between the number and unit was removed from “50°C” (line 81) to maintain consistency in the manuscript.*

**L190, L227, L243, L257: Please remove the underlining from the titles and revise them to conform to the journal’s standard format for chapters and section headings.**

*Thanks for highlighting the formatting issue. The titles “Spectral characteristic and chemical groups” (line 197), “Wettability” (line 234), “Surface roughness” (line 250), and “Elasticity” (line 264) under the “Result” section were updated according to the journal’s guidelines (Bold type, 16pt font).*

*When revising the journal’s guidelines, the table and figure legends were also updated according to the journal`s guidelines. (lines 63, 110, 134-135, 205, 221, 226-227, 247-248, 260-261, and 274.*

**L193: Please use an en dash for numerical ranges and insert commas for numbers at the thousand level.**

*We follow this recommendation (see comments above), and the manuscript has been updated consistently.*

**L270: The paragraphs in the discussion section are excessively long. For better readability, please divide them into shorter paragraphs based on their logical and thematic structure.**

*We agree with the reviewer. To increase clarity and readability, the discussion was divided into shorter and thematically structured paragraphs. The discussion was broken into paragraphs on lines 286, 292, 299, 329, and 345.*

**L341: The conclusion should more clearly summarize the main findings of the study. In addition, it would be beneficial to explicitly mention the limitations of the current work and to suggest directions for future research. Please revise this section from that perspective.**

*To improve clarity and highlight the limitations of our study, we add the following text in the conclusion:*

*Line 363-367: “Even though the UV radiation exposure time for all films was less than the designated lifetime indicated by the manufacturer,* *our results show clear evidence of photodegradation on the different agricultural plastics used in this study, as well as an accelerating effect of abrasion on the degradation process. Additionally, we found indications of potential microplastic formation...”*

*Line 375-378: “Future laboratory-based research should consider other environmental variables also important for degradation processes that were not considered in this study, such as humidity and temperature. A logical next step is to monitor UV-induced film degradation under real-world field conditions.”*

**L373: Overall, the number of references cited in the manuscript is relatively small. Please consider strengthening the literature review by incorporating additional relevant and up-to-date references.**

*We added five new, relevant, and recently published references to the manuscript. The following changes were made:*

*On lines 312-316: “The use of additives* *mainly controls degradation processes.* *Nevertheless, the different concentrations and types of additives will have different effects, as the combination of different polymer types and additives present different sensitivities, influencing the degradation mechanisms (35). As shown in this study, films with similar purposes may behave differently (Fig 3).”*

*On line 321: “…and Yang et al. (37).”*

*On lines 321-322: “Additionally, in line with our results, Liang et al. (38) also reported higher wettability on samples aged under soil burial”*

*On lines 339:* *“…with thinner films presenting a higher degradation rate* *(39, 40).”*

*35 Niu L, Shen J, Li Y, Chen Y, Zhang W, Wang L. Plastic additives alter the influence of photodegradation on biodegradation of polyethylene/polypropylene polymers in natural rivers. Journal of Hazardous Materials. 2025;489:137542.*

*37 Yang J, Song K, Tu C, Li L, Feng Y, Li R, et al. Distribution and weathering characteristics of microplastics in paddy soils following long-term mulching: A field study in Southwest China. Science of the Total Environment. 2023;858(Pt 2):159774.*

*38. Liang J, Chen X, Duan X, Gu X, Zhao X, Zha S, et al. Natural aging and adsorption/desorption behaviors of polyethylene mulch films: Roles of film types and exposure patterns. Journal of Hazardous Materials. 2024;466:133588.*

*39. Xiong XB, Zhao ZY, Wang PY, Zhou R, Cao J, Wang J, et al. In situ degradation of low-density polyethylene film in irrigation maize field: Thickness-dependent effect. Science of the Total Environment. 2023;858(Pt 3):159999.*

*40. Uzamurera AG, Wang PY, Zhao ZY, Tao XP, Zhou R, Wang WY, et al. Thickness-dependent release of microplastics and phthalic acid esters from polythene and biodegradable residual films in agricultural soils and its related productivity effects. Journal of Hazardous Materials. 2023;448:130897.*

Point-by-point response – Reviewer 2

**1. The manuscript repeatedly refers to “microplastic formation” and “release of microplastic particles,” yet no direct quantification or identification of microplastics (e.g., particle counting, size distribution, mass balance) is performed. The conclusions are instead inferred from changes in surface roughness, wettability, and FTIR indices. While this interpretation is reasonable, the wording in the Discussion and Conclusion sections should be softened. Statements implying confirmed microplastic generation should be reframed as potential or indirect evidence of microplastic formation unless supported by direct measurements.**

*To clarify this, the following changes were made in the manuscript:*

*In the Abstract: “…and indications for the removal of microplastic…”*

*On line 338–339: “…Hence, UV light and abrasion are interacting processes that can accelerate microplastic formation…”*

*On line 370–371: “This study indicates that microplastic formation occurs…”*

**2. The abrasion treatment was performed without replicates (61 days, single vessel per treatment). Given the variability inherent to abrasion processes, this limits the statistical robustness of the results, particularly for surface roughness and FTIR-based indices. This limitation should be explicitly acknowledged in the Methods and Discussion sections. The authors may also consider clarifying why replication was not feasible and how this might affect result interpretation.**

*We agree that for the surface roughness performed using the LSM, the limited number of samples can be an issue. The number of replicates was limited due to the UV chamber capacity. However, we were interested in changes across the entire surface area. Therefore, the analysis aimed to determine the average surface roughness to compare between treatments. Hence, the LSM provides surface roughness information that integrates the entire sample surface and, therefore, the most representative mean.*

*For the FTIR analysis, we collected three spectra per sample, providing information on the variability of the samples tested and the minimum number for statistical tests.*

*To make it clear in the manuscript regarding the statistical limitations, the following changes were made:*

*On line 92: “**Due to the limited number of samples, after the UV treatment, one sample of each mulch film was divided into two 2 cm x 2 cm pieces.”*

*On lines 161-164: “Thereby, average surface roughness was determined using LSM, which integrates roughness across the full sample area to yield a representative mean for each treatment. This representative mean was prioritised over additional replicates of smaller areas.”*

**3. The manuscript attempts to relate artificial UV exposure to natural sunlight exposure using a generalized energy conversion approach. While helpful, this conversion remains approximate and does not account for additional environmental factors such as temperature fluctuations, moisture, or soil-film contact. The Discussion would benefit from a clearer statement that the laboratory exposure represents a simplified scenario and may not fully capture field-scale degradation dynamics.**

*We fully agree that our experimental design focuses only on UV radiation, not accounting for all the variability in real environmental exposure. We highlighted this on lines 81–82 (**“No other parameter was controlled.”), however, we changed our manuscript to closely point out this limitation:*

*On lines 352–353: “…as weathering could be influenced by other factors such as temperature and humidity…”*

*On lines 375-378: “Future laboratory-based research should consider other environmental variables also important for degradation processes that were not considered in this study, such as humidity and temperature. A logical next step is to monitor UV-induced film degradation under real-world field conditions.”*

**4.The conclusion that no increased fragmentation potential exists is based on unchanged Young’s modulus values within the applied UV exposure range. However, fragmentation in real agricultural systems is influenced not only by elastic modulus but also by embrittlement, crack initiation, and handling stresses. The authors should clarify that the absence of elasticity changes under the tested conditions does not necessarily rule out fragmentation under longer exposure times or combined environmental stresses.**

*We agree that UV is not the only factor responsible for the degradation of agricultural plastic. To clarify that different and longer exposure factors could still affect the fragmentation of the mulch and greenhouse films in different ways, the following change was made:*

*On lines 355–358: “However, the lack of substantial change in film elasticity in this study does not necessarily exclude fragmentation of mulch and greenhouse films over longer exposure times and/or combined environmental factors. It only highlights that the parameters used in this study were insufficient to cause fragmentation.”*

**5.The terms “macroplastic residues,” “plastic particles,” and “microplastic formation” are sometimes used interchangeably. A short clarification of terminology early in the manuscript would improve readability.**

*For clarification, we defined the terms “macroplastic” and “microplastic” and removed the term “plastic particles” from the manuscript. The following changes were made:*

*Line 23: “…may lead to the detachment of residues during mulch application…”*

*Line 25: “…macroplastic (> 5 mm)...”*

*Line 316-317: “…microplastics (< 5 mm)…”*

*Line 325: “…detachment of microplastics…”*

**6. The manuscript notes that differences between films may arise from undisclosed additives. While this is reasonable, a brief discussion of how this uncertainty affects comparability between film types would strengthen the interpretation.**

*We appreciate the comment, and to enrich our discussion regarding the influence of additives, the following change was made:*

*On line 312–316:* *“**The use of additives is crucial for the degradation processes. Nevertheless, the different concentrations and types of additives will have distinct effects, as the combination of different polymer types and additives may present different sensitivities, influencing the degradation mechanisms (35). As shown in this study, films with similar purposes may react differently (Fig 3)”*
